# Significant association of serum creatinine with HbA1C in impaired glucose tolerant Pakistani subjects

**DOI:** 10.12669/pjms.314.7063

**Published:** 2015

**Authors:** Tasnim Farasat, Saima Sharif, Shagufta Naz, Sabiha Fazal

**Affiliations:** 1 Prof. Dr. Tasnim Farasat, Ph.D. Department of Zoology, Lahore College for Women University, Jail Road, Lahore, Pakistan; 2 Dr. Saima Sharif, Ph.D. Assistant Professor, Department of Zoology, Lahore College for Women University, Jail Road, Lahore, Pakistan; 3 Dr. Shagufta Naz, Ph.D. Assistant Professor, Department of Zoology, Lahore College for Women University, Jail Road, Lahore, Pakistan; 4 Prof. Sabiha Fazal, Department of Zoology, Lahore College for Women University, Jail Road, Lahore, Pakistan

**Keywords:** IGT, HbA1c, Serum creatinine, Oral Glucose Tolerance Test (OGTT)

## Abstract

**Objective:**

The present study was conducted to assess the serum concentration of creatinine and determine its relationship with potential risk factors of diabetes in Impaired Glucose tolerance subjects.

**Methods:**

This cross sectional study was conducted on 100 IGT patients who attended Amin Hayat diabetic center in Lahore from January 2011- June 2011. Patients with age group 34-67 years, (both sexes) were included in the study. Different demographic parameters as age, BMI, WHR, B.P, personal history and socioeconomic status were recorded. Oral Glucose Tolerance Test was performed. The biochemical parameters including HbA1c, lipid profile, urea, uric acid, creatinine and bilirubin level were measured by chemistry analyzer.

**Results:**

A strong correlation between creatinine and HbA1c was observed. The level of creatinine was also significantly associated with age in IGT subjects. Creatinine is non-significantly correlated with Cholesterol, LDL-Chol and TG while negatively significantly associated with BMI, fasting blood glucose and HDL-Chol.

**Conclusion:**

The present study concluded significant association of serum creatinine with HbA1c, BMI and HDL cholesterol.

## INTRODUCTION

Impaired glucose tolerance (IGT) form an intermediate stage in the natural history of diabetes mellitus. Patients with IGT have a significant risk of developing diabetes and thus are an important target group for primary prevention.^1^ The transition from the early metabolic abnormalities that precede IGT to diabetes may take many years; however, current estimates indicate that most individuals (up to 70%) with these pre-diabetic states eventually develop diabetes.^2^

Information about the natural history and pathogenesis of diabetes indicates that this disease has a prolonged prediabetic phase. Obesity (especially abdominal obesity) is an important feature of the IR syndrome, which may lead to IGT and T2DM.^3^

Skeletal muscle is the most important site of insulin resistance and accounts for approximately 90% of overall glucose disposal after glucose infusion. Creatinine is the only metabolite of creatin which is mainly (98%) located in striated muscle.^4^ Since serum creatinine is highly correlated with 24-h urine excretion in subjects with normal renal function,^5^ Low serum creatinine levels were associated with a higher risk of T2DM in a recent study of non-obese middle- aged Japanese men,^6^ it is speculated that low creatinine might reflect low muscle mass volume.

HbA1c is a measure of erythrocyte hemoglobinglycation since erythrocytes have about 120 days life span, and reflects overall blood glucose levels over a period of 2-3 months and further, used to monitor diabetic treatment. It has been recognized that the HbA1c as an essential adjunct to regular self-blood glucose measurement assisting in the achievement of the best possible glycemic control. Renal failure can have complex influences on HbA1c formation and measurements. The reason being the fact that urea–derived isocyanate can lead to the formation of carboxylated Hb, which can be indistinguishable from HbA1c, when using some glycated Hemoglobin methods.^7^

Relationship between HbA1c and serum creatinine is not clear in impaired glucose tolerant subjects. This study was aimed to find out the relationship of serum creatinine with BMI, glycemic levels, HbA1c and lipid profile.

## METHODS

The study population consisted of 100 IGT subjects. Sample size was calculated by using sample calculator on Raosoft with 95% confidence level, 9% margin of error and taking expected response distribution as 70%. Samples were collected from people suffering from IGT who attended Amin Hayat Memorial Hospital in Lahore from January 2011 to June 2011. All the subjects aged from 34 to 67 years old (mean age 50.81 ± 0.97 years), were enrolled. IGT was defined as a fasting glucose level of 100-125 mg/dL or as a random glucose level of 140-199 mg/dL diagnosed by the hospital management.^8^ The study protocol was approved by the Ethics Committee of Board of Directors, Hamza Foundation at Amin Hayat Memorial Medical Center, Lahore. Blood samples were collected and serum was separated and stored in refrigerator at -70°C until analysis. An oral glucose tolerance test (OGTT) was conducted after a 12 h overnight fast. In all 75g of glucose solution was ingested after fasting blood sample was obtained for plasma glucose. Blood samples were obtained at baseline, 30 minutes, 60 minutes and 120 minutes after ingestion and were later assayed for glucose concentrations. Then subjects were classified in two groups according to glucose tolerance status as defined by the American Diabetes Association (ADA) criteria.^8^

Normal glucose tolerance (NGT) was defined as fasting plasma glucose (FPG) of less than 5.5 mmol/l (100 mg/dl) and 2-h postload glucose of <7.8 mmol/l (140 mg/dl).

Prediabetes was defined as FPG of 5.5-6.9 mmol/l (100-125 mg/dl) and/or impaired glucose tolerance (IGT) (2-h postload glucose of 7.8-11.0 mmol/l [140-199 mg/dl]).

The data regarding age, sex, height, weight, body mass index (BMI), waist, hip, and waist hip ratio, systolic and diastolic blood pressure (B.P) was collected. BMI was calculated as weight divided by the square of height. All biochemical determinations were carried out using the standard laboratory methods. HbA1c, serum cholesterol, high density lipoprotein (HDL)-Chol, low density lipoprotein (LDL)-Chol, triglycerides (TG), urea, uric acid and creatinine were assessed by using an automatic chemistry analyzer. Descriptive statistical analysis of all the studied variables was done and Pearson’s correlation coefficient was applied to observe the relationship between serum creatinine and studied clinical parameters using SPSS software version 13.0 (ILO, Chicago).

## RESULTS

The demographic and biochemical characteristics of study population are presented in Table-I.

**Table-I:** Demographic and biochemical characteristics of IGT subjects.

Parameters	Mean	Std.
Age (years)	49.29 ± 0.72	7.25
Height (cm)	155.37 ± 0.84	8.40
Weight (Kg)	77.07 ± 1.46	14.58
BMI (Kg/m²)	31.26 ± 0.61	6.04
Waist (cm)	71.57 ± 3.59	35.80
Hip (cm)	71.69 ± 3.38	33.67
WHR	0.98 ± 0.006	0.06
Fasting blood glucose (mg/dL)	101.22 ± 1.32	13.15
Random blood glucose (mg/dL)	186.98 ± 0.63	6.25
60 minutes	167.36 ± 1.44	14.35
120 minutes	141.21 ± 1.58	15.77
HbA1c (%)	6.12 ± 0.071	0.71
Systolic B.P (mm Hg)	127.88 ± 0.74	7.39
Diastolic B.P (mm Hg)	87.30 ± 0.97	9.70
Cholesterol (mg/dL)	192.80 ± 3.86	38.41
HDL-Chol (mg/dL)	38.93 ± 0.59	5.96
LDL-Chol (mg/dL)	120.40 ± 3.51	34.89
TG (mg/dL)	166.19 ± 6.11	60.81
Urea (mg/dL)	22.87 ± 1.06	10.55
UA (%)	5.42 ± 0.13	1.32
Creatinine (mg/dL)	0.83 ± 0.02	0.22

### Correlation Analysis

**Fig.1:**
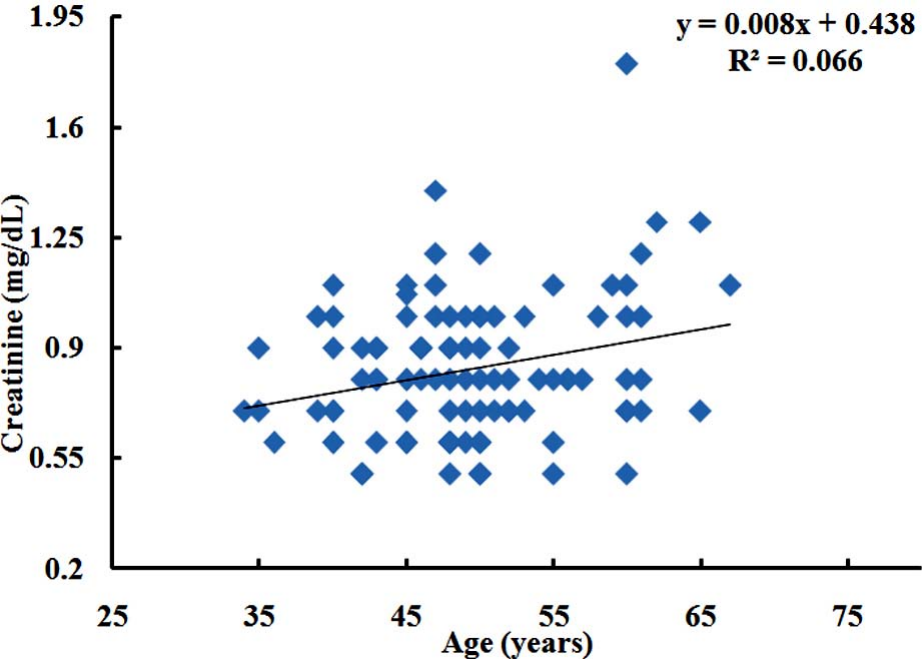
Correlation of creatinine (mg/dL) age (years) in IGT subjects.

**Fig.2:**
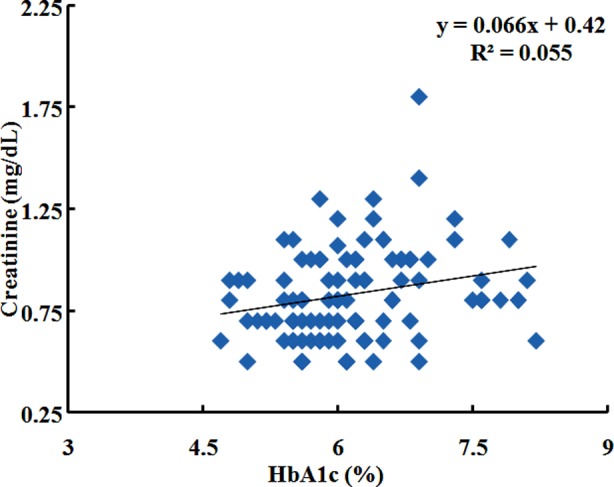
Correlation of Creatinine (mg/dL) with HbA1c % in IGT subjects.

Creatinine was significantly correlated with age(r = 0.257, P ≤ 0.05) (Fig.1). It is negatively correlated with BMI (Kg/m²) (r = -0.243, P ≤ 0.05), fasting blood glucose(r = -0.011, P ≥ 0.05). Blood glucose level at 120 min (r = 0.028, P ≥ 0.05) shows no significant correlation. Creatinine is significantly correlated with HbA1c (%) (r = 0.236, P ≥ 0.05) (Fig.2) (Table-IIa). Creatinine was non-significantly correlated with Chol (r = 0.007, P ≥ 0.05). The relationship with HDL (r = -0.208, P ≤ 0.05) was inverse but significant. Relationship of creatinine with LDL (r = -0.004, P ≥ 0.05), TG (r = -0.038, P ≥ 0.05) was not significant (Table-IIb).

**Table-IIa:** Correlation of Creatinine (mg/dL) with Age (years), BMI (Kg/m²), Fasting and Random Blood Glucose (mg/dL), HbA1c (%).

	Age (years)	BMI (Kg/m²)	Fasting glucose (mg/dL)	Random blood glucose (mg/dL) at 120 min	HbA1c (%)
Creatinine (mg/dL)	r = 0.257 P ≤ 0.05	r = -0.243 P ≤ 0.05	r = -0.011 P ≥ 0.05	r = 0.028 P ≥ 0.05	r = 0.236 P ≤ 0.05

**Table-IIb:** Correlation of creatinine with Cholesterol, HDL-Cholesterol, LDL-Cholesterol, TG.

	Chol (mg/dL)	HDL (mg/dL)	LDL (mg/dL)	TG (mg/dL)
Creatinine (mg/dL)	r = 0.007 P ≥ 0.05	r = -0.208 P ≤ 0.05	r = -0.004 P ≥ 0.05	r = -0.038 P ≥ 0.05

## DISCUSSION

In the present study, the relationship of serum creatinine was observed with other metabolic parameters that are known risk factors for IGT including obesity and lipid profile. Subjects with IGT have been shown to be abdominally obese. Serum creatinine concentration is simple cheap and widely used for evaluation of renal function and is a risk factor of diabetes.^6^

BMI negatively was associated with creatinine in this study. Results are not in accordance with study by Banfi and Fabbro^9^ who reported significant association between BMI and creatinine concentrations. This may be due to higher visceral fat and metabolically healthy subjects in our population. The present study revealed significant relationship between serum creatinine and age. The results are in accordance with a study conducted by Sheikh and his colleagues in 2009.^10^ A contradiction was found by Musch and his colleagues in 2006^11^ who revealed no significant association. This may be due to ethnicity differences. A significant association was observed between HbA1c and creatinine. It is reported that in both genders serum creatinine is significantly associated with different categories of impaired glucose regulation independent of known metabolic risk factors.

The IGT state is also associated with alterations in plasma lipoprotein-lipid concentrations^12^, including the presence of small dense LDL particles.^3^ Hypertension and high concentrations of free fatty acids and low total bilirubin had also been reported in subjects with IGT.^13^

In our study other biochemical parameters such as BMI, fasting blood glucose after 30, 60, and 120 minutes, were significantly higher in prediabetes and significantly associated with serum creatinine. Although serum total cholesterol were in normal range but low level of HDL in our population were observed that may be due to disturbance of lipid metabolism which appears to be an early event in the development of type 2 diabetes potentially preceding the disease by several years.^14^ We did not find significant relationship of serum urea and uric acid with HbA1c in prediabetes contrary to other studies.^15^

## CONCLUSION

Serum creatinine is significantly associated with different categories of glucose regulation independent of known metabolic risk factors and life style factors. The association between creatinine level and HbA1c can be useful predictor in diabetes.
